# Unusual clinical features associated with congenital generalized lipodystrophy type 4 in a patient with a novel E211X *CAVIN1* gene variant

**DOI:** 10.1186/s40842-020-00095-3

**Published:** 2020-05-14

**Authors:** Ekaterina Sorkina, Polina Makarova, Liubov Bolotskaya, Irina Ulyanova, Tatyana Chernova, Anatoly Tiulpakov

**Affiliations:** Endocrinology Research Centre, 11, ulitsa Dmitriya Ulianova, Moscow, Russian Federation 117036 Russia

**Keywords:** Congenital generalized lipodystrophy type 4, *CAVIN1*, Insulin dependent diabetes mellitus, Vitamin D deficiency, Bilateral cataracts

## Abstract

**Background:**

Congenital generalized lipodystrophy (CGL) is a rare disorder characterized by the lack of adipose tissue and metabolic complications with predominantly autosomal recessive inheritance. There are 6 different genes known to cause CGL with 4 main types recognized to date, which differ by the degree of fat loss, association with mental retardation and metabolic disorders, with CGL type 1 and 2 being the most common. Twenty seven cases of СGL type 4 from Japan, Oman, UK, Turkey, Mexico, Saudi Arabia, USA were reported previously. This report details our clinical experience with the first patient from Russia with CGL type 4.

**Case presentation:**

A 36-year-old patient, who has been suffering from generalized lipoatrophy since the first months of life and myopathy and gastrointestinal dysmotility since early childhood, developed dysmenorrhea and diabetes mellitus at the age of 19, bilateral cataracts when she was only 22 y.o., osteoporosis with vitamin D deficiency and hypocalcemia at the age of 28, diabetic foot syndrome and hyperuricemia when she was 35 y.o. Sequencing of lipodystrophy candidate genes detected a novel pathogenic homozygous variant p.631G < T: p.E211X in the *CAVIN1* gene, confirming the diagnosis of CGL type 4.

**Conclusions:**

In comparison with previously reported patients with CGL type 4, our patient has diabetes mellitus, vitamin D deficiency, hypocalcemia, bilateral cataracts and hyperuricemia. All these manifestations are known to be associated with other lipodystrophy syndromes, but to our knowledge it is the first time they have been reported to be associated with CGL type 4.

## Background

Congenital generalized lipodystrophy (CGL) is a rare disorder characterized by the lack of adipose tissue and metabolic complications with predominantly autosomal recessive inheritance. There are 6 different genes known to cause CGL [1] with 4 main types recognized to date, which differ by the degree of fat loss, association with mental retardation and metabolic disorders, with CGL type 1 and 2 being the most common ones [2]. The general prevalence of CGL is about 0.1: 100,000 of population, with certain exceptions: Brazilians (the state of Rio Grande do Norte, Brazil, has one of the highest CGL prevalence rates worldwide, up to 498:100,000) [3], Lebanese 0.5:100,000; Portuguese 0,2:100,000; Omanis 4:100,000 [4]. Currently there are only 27 patients from 20 families reported to have CGL type 4. In this article we have reported an unusual clinical presentation of the first Russian patient with CGL type 4.

## Case presentation

A 36-year-old female patient of Tatarian origin was referred to the Endocrinology Research Centre, Moscow, with a diagnosis of type 1 diabetes mellitus complicated by diabetic foot syndrome. According to the medical documents, generalized lipoatrophy developed during the first months of life, and from the early childhood she suffered from an umbilical hernia, muscular hypotonia and delayed motor development (without mental retardation). Later moderate amyotrophy of the muscles of the shoulder girdle developed, with a lack of reflexes in the legs, muscle weakness and myalgia of lower extremities. Between the ages of 17 and 20 the patient underwent multiple surgeries for dolichosigmoid, perforation of diverticula and peritonitis, which resulted in local muscular weakness and malabsorption leading to generalized muscular weakness and dysphagia. At 19 years of age diabetes mellitus type 1 was diagnosed with normal body weight but for 2 years the patient was well compensated by a diet with fast carbohydrates restriction. At the age of 21, she started insulin therapy. A wound defect (trophic ulcer) in the area of the inner surface of the right thigh was diagnosed at the age of 35; after a few months, diabetic neuro-osteoarthropathy of the right foot was also diagnosed. For 3 months, the patient used a Total Contact Cast with positive effects. The patient had amenorrhea with 2 episodes of menorrhagia in her life between the ages of 18 and 19. Bilateral cataracts were first diagnosed at the age of 22, and were treated surgically 12 years later. Marked diffuse osteoporosis was diagnosed at the age of 28 by a multi-layer spiral CT (Computed Tomography) scan.

### Physical examination

She had acromegaloid features, phlebomegaly and hypertrophy of the skeletal muscles in the upper and lower extremities, muscular hypotonia, generalized lipoatrophy, but no signs of hirsutism and clitoromegaly were found. Her height was 160 cm, weight 46.8 kg, BMI (Body mass index) 18.1 kg/m^2^.

### Diagnostic tests

**Fig. 1:**
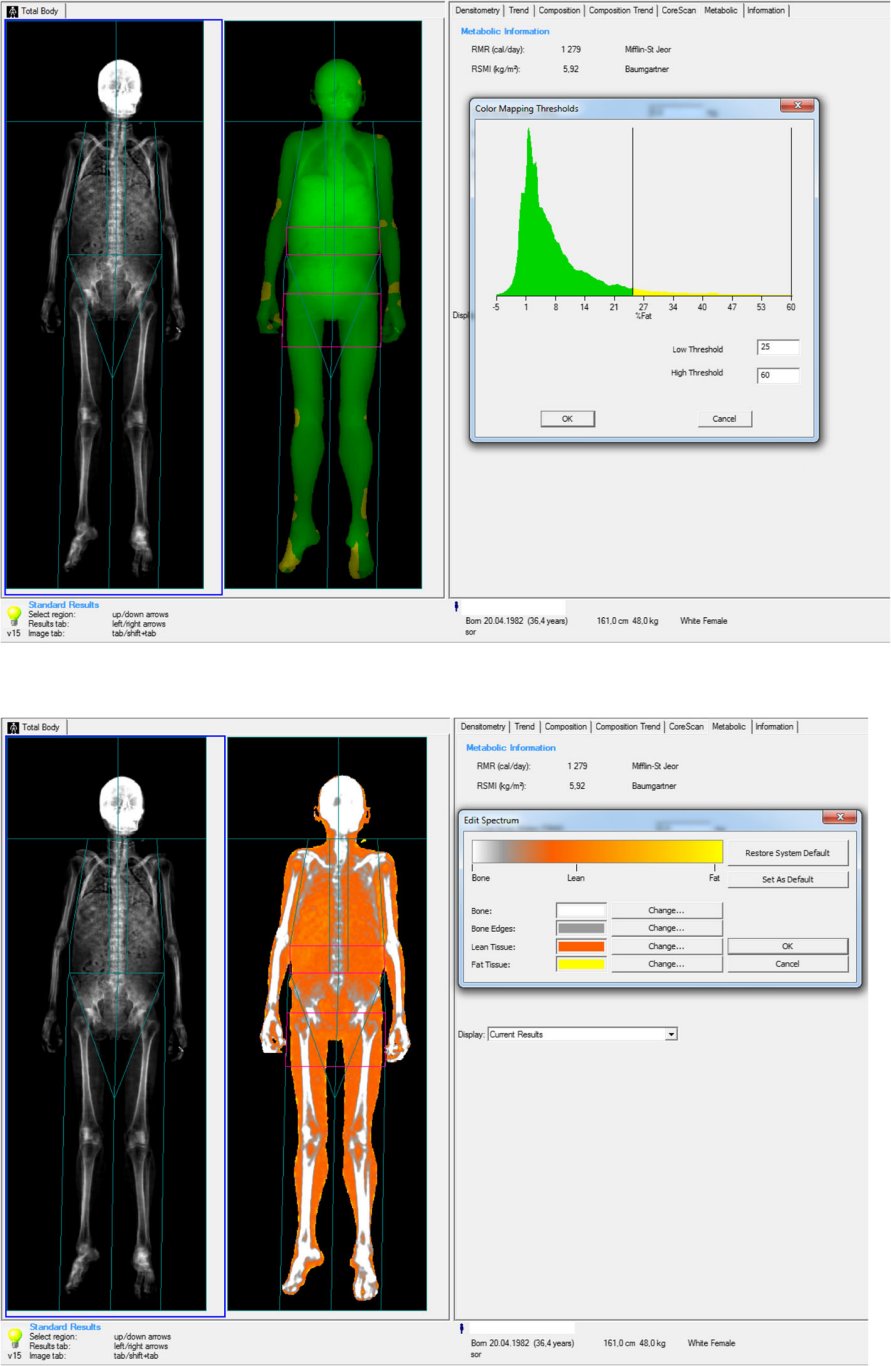
“Total body” densitometry

Skin fold measurements, impedancemetry and “Total body” densitometry showed a significant decrease of subcutaneous fat tissue. The skin fold measurements were as following: supraclavicular skin fold, 4 mm; subscapular skin fold, 6 mm; triceps skin fold, 4 mm; anterior surface of the abdomen skin fold, 4 mm; hip skin fold, 6 mm; anterior surface of the thigh skin fold, 6 mm; posterior surface of the tibia skin fold, 3 mm. Impedancemetry showed 11.9% of body fat, and “Total body” densitometry demonstrated 7.8% of total fat (Fig. 1). An abdominal ultrasound showed hepatosplenomegaly with severe hepatic steatosis. There was no cardiac pathology, except sinus tachycardia shown in the electrocardiogram. The densitometry showed severe osteoporosis of the lumbar spine (T-score L1-L4: − 5.6), osteoporosis of the proximal femur (T-score Neck: − 5.4). The laboratory data is presented in Table 1.

**Table 1:** Laboratory data (Blood. Age 36 years)

Variables	Values	Normal range
Fasting glucose, mmol/l	8.8	3.1–6.1
HbA1c, %	7.3	4–6
C-peptide, ng/ml	0.928	1.1–4.4
Anti-tyrosine phosphatase antibodies, U/ml	0.144	neg. < 8
Anti-islet cell antibodies, U/ml	0.58	neg. < 0.95
Anti-insulin antibodies, U/ml	17	pos. > 10
Zinc transporter 8 antibodies, U/ml	1.436	neg. < 15
Anti-GAD antibodies, U/ml	0.7	neg. < 1
Total cholesterol, mmol/l	5.55	3.3–5.2
HDL cholestrol, mmol/l	0.452	1.15–2.6
LDL cholestrol, mmol/l	1.435	1.1–3
Triglycerides, mmol/l	8.14	0.1–1.7
Leptin, ng/ml	5.382	3.7–11.1
Adiponectin, ng/ml	2.54	8.2–19
Creatinine kinase, U/l	472	29–168
ALT, U/l	42	0–55
AST, U/l	75	5–34
GGT, U / l,	139	9–36
Calcium, mmol/l	2.28	2.15–2.55
Ionized calcium, mmol/l	0.9	1.03–1.29
Vitamin D, ng / ml	27.2	> 30
Creatinine, mcmol/l	69.2	50–98
CKD – EPI, ml/min/1,73 m^2^	98	90–120
Urea, mmol/l	9.4	2.5–6.7
Uric acid, μmol	444.25	142–339

*HbA1c* Hemoglobin A1c, *GAD* Glutamic acid decarboxylase, *HDL* High-density lipoprotein, *LDL* Low-density lipoprotein, *ALT* Alanine aminotransferase, *AST* Aspartate aminotransferase, *GGT* Gamma-glutamyl transpeptidase, *CKD – EPI* Chronic Kidney Disease Epidemiology Collaboration

### Family history

**Fig. 2:**
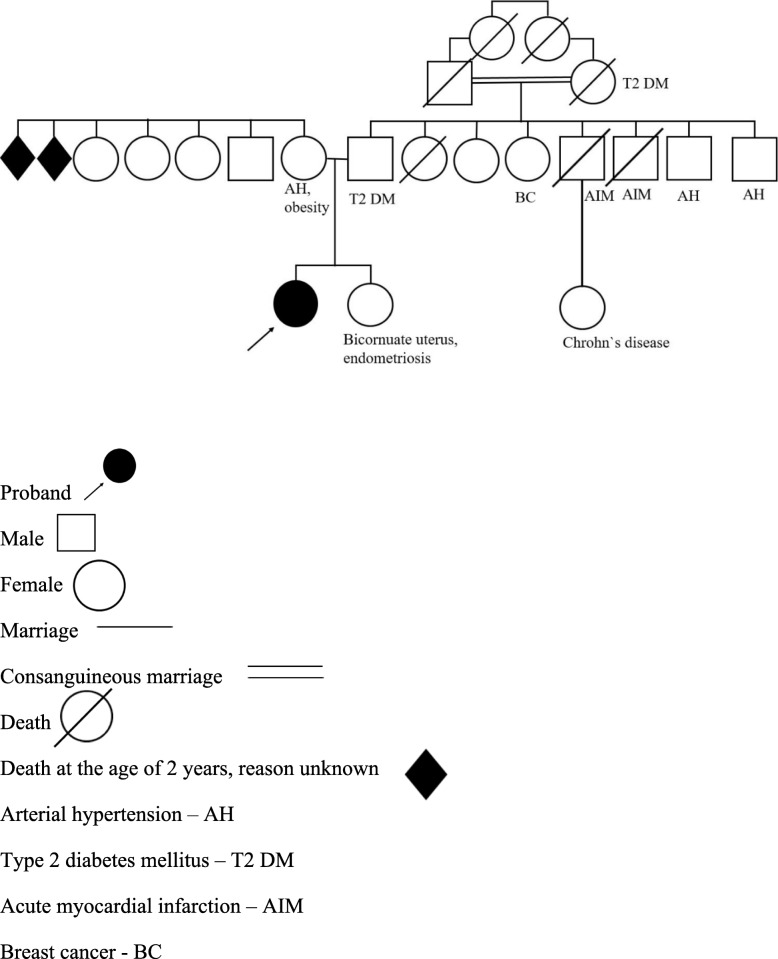
Genealogical tree

(Fig. 2): a consanguineous marriage of grandparents (cousins) from the father’s side, type 2 diabetes (father and grandmother from the father’s side), breast cancer (father’s sister), acute myocardial infarction (2 father’s brothers), arterial hypertension (2 father’s brothers), bicornuate uterus and endometriosis (sister), Crohn’s disease (cousin from the father’s side), arterial hypertension and obesity (mother), death in early childhood from the unknown reason (mother’s brother and sister). The patient reported that the grandmother from the father’s side had a short stature.

The patient received insulin therapy (insulin glargine 20 U/day, insulin glulisine 40 U/day), nephroprotective therapy (enalapril 2.5 mg/day), bisoprolol 5 mg/day for tachycardia, antihyperuricemic therapy (allopurinol 150 mg/day), the native form of vitamin D, alfacalcidol and calcium for osteoporosis. The patient also receives symptomatic therapy for gastrointestinal pathology and nutritional therapy.

**Fig. 3:**
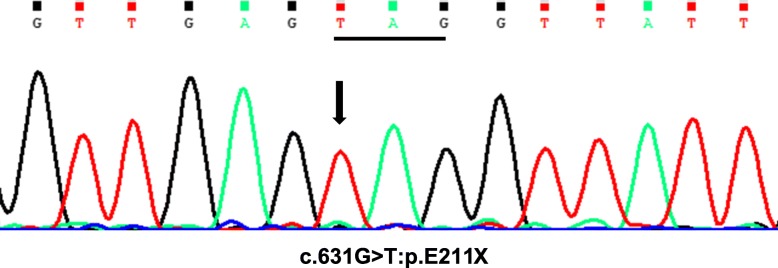
Electropherogram of DNA sequence of the *CAVIN1* gene showing a homozygous variant c.631G < T resulting in the p.E211X mutation (codon is underlined) in the patient

Based on typical clinical signs of generalized lipodystrophy (total lipoatrophy, muscular hypertrophy, phlebomegaly, acromegaloid features, hypertriglyceridemia, hepatosplenomegaly, steatohepatitis) and early onset of the disease, CGL was suspected. Taking into account muscular pathology, CGL type 4 appeared probable. Due to the variety of clinical features of different types of lipodystrophy syndromes and progeroid syndromes sequencing of 18 lipodystrophy candidate genes (*AGPAT2, BSCL2, CAV1, CAVIN1, PSMB8, LMNA, PPARG, PLIN1, AKT2, CIDEC, LIPE, LMNB2, PIK3CA, PPP1R3A, POLD1, WRN, ZMPSTE24, BANF1*) using a custom Ion Ampliseq panel and Personal Genome Machine (ThermoFisher Scientific, Waltham, MA, USA) semiconductor sequencer (Ion Torrent) was performed. A novel pathogenic homozygous variant c.631G < T: p.E211X was detected in the *CAVIN1* gene (Fig. 3), confirming the diagnosis of CGL type 4.

## Discussion

All types of CGL are characterized by a near-complete lack of fat starting at birth or infancy, prominent muscles, phlebomegaly, hepatomegaly, umbilical prominence and a voracious appetite in childhood. Genetic and phenotypic heterogeneity is well documented in patients with CGL, as well as the overlap of clinical findings in different types of CGL.

CGL type 4 is a unique form of generalized lipodystrophy characterized by all the symptoms listed above as well as myopathy, cardiac arrhythmias, skeletal abnormalities and gastrointestinal dysmotility (Tables 2 and 3). CGL type 4 is usually associated with metabolic abnormalities secondary to insulin resistance, however acanthosis nigricans has been reported in only 2 patients out of 27 described patients with CGL type 4.

**Table 2:** Clinical Features of Patients With CGL type 4 as a Result of Different *CAVIN1* Mutations (Patient 1 – described patient, patients 2–28 – previously described patients [5–14])

Clinical features/Patients	1	2	3	4	5	6	7	8 (s.9)	9 (s.8)	10	11 (s.12)	12 (s.11)	13	14	15	16	17	18 (s.19, 20)	19 (s.18, 20)	20 (s.18, 19)	21	22	23 (s.24, 25)	24 (s.23, 25)	25 (s.23, 24)	26	27 (s. 26)	28
Country of origin	R	UK	J	J	J	J	J	M	M	T	M	M	T	J	J	O	O	O	O	O	O	O	O	O	O	SA	SA	U
Age at report, y	36	13	8	14	10	27	24	11	1,4	11	12	7	13	3	3 y. 11 m.	14	2	15	11	3	11 m	1	14	10	4	6	3	15
Age of onset of lipodistrophy, y	36	NA	NA	NA	NA	NA	NA	NA	NA	NA	NA	NA	NA	2.5	NA	14	NA	NA	NA	NA	NA	NA	NA	NA	NA	NA	NA	NA
Sex	F	F	F	F	M	M	M	M	F	F	F	M	F	F	M	M	NA	NA	NA	NA	NA	NA	NA	NA	NA	M	M	M
Generalized lipodistrophy	Y	Y	Y	Y	Y	Y	Y	Y	Y	Y	Y	Y	Y	Y	Y	Y	Y	Y	Y	Y	Y	Y	Y	Y	Y	Y	Y	Y
Skeletal muscle hypertrophy	Y	Y	Y	NA	NA	Y	Y	Y	Y	Y	Y	Y	Y	N	Y	Y	NA	NA	NA	NA	NA	Y	Y	Y	Y	Y	Y	N
Muscle weakness	Y	Y	Y	NA	NA	Y	Y	Y	N	NA	Y	Y	NA	N	Y	Y	NA	Y	Y	Y	NA	NA	Y	Y	Y	N	N	Y
Muscle pain	Y	Y	NA	Y	NA	NA	NA	N	N	NA	Y	NA	NA	N	N	Y	NA	NA	NA	NA	NA	NA	NA	NA	NA	N	N	N
Acanthosis nigricans	N	N	N	N	N	N	N	Y	N	N	Y	NA	N	N	NA	N	NA	NA	NA	NA	NA	N	N	N	N	N	N	Y
Rigid spine or scoliosis	Y	Y	Y	N	N	Y	Y	N	NA	Y	NA	NA	Y	N	N	Y	NA	NA	NA	NA	NA	N	Y	NA	NA	N	N	N
Hypertrophic pyloric stenosis	N	N	NA	NA	NA	NA	NA	Y	Y	NA	NA	NA	NA	NA	N	Y	Y	Y	Y	Y	N	Y	Y	Y	Y	N	N	N
Acromegaloid features	Y	Y	N	NA	NA	Y	Y	NA	N	NA	Y	NA	Y	NA	NA	Y	NA	NA	NA	NA	NA	N	NA	NA	NA	Y	N	N
Joint contractures	N	Y	Y	N	N	Y	NA	Y	N	NA	NA	NA	NA	N	NA	Y	NA	NA	NA	NA	NA	Y	Y	Y	Y	Y	N	Y
Atlantoaxial instability	NA	Y	NA	NA	NA	NA	NA	Y	NA	NA	Y	Y	Y	NA	NA	NA	NA	NA	NA	NA	NA	NA	NA	NA	NA	N	N	Y
Arrhythmia	N	Y	Y	N	N	Y	N	NA	N	NA	N	NA	N	N	N	Y	NA	NA	NA	NA	NA	NA	NA	NA	NA	N	N	N
Tachycardia	Y	Y	N	N	N	N	N	NA	N	NA	N	NA	Y	N	N	Y	NA	NA	NA	NA	NA	NA	NA	NA	NA	N	N	Y
Long-QT syndrome	N	Y	N	N	N	N	N	NA	N	NA	N	NA	N	N	N	Y	NA	NA	NA	NA	NA	NA	NA	NA	NA	N	N	N
Diabetes mellitus	Y	N	N	N	N	N	N	N	N	N	N	N	N	N	N	N	NA	NA	NA	NA	NA	NA	NA	NA	NA	N	N	N
Neuropathy	Y	N	NA	NA	NA	NA	NA	NA	NA	NA	NA	NA	NA	NA	N	Y	NA	NA	NA	NA	NA	NA	NA	NA	NA	N	N	N
Dysphagia	Y	Y	NA	NA	NA	NA	NA	Y	Y	NA	Y	Y	NA	NA	NA	Y	NA	NA	NA	NA	NA	NA	NA	NA	NA	N	N	N
Motor development delay	Y	N	N	N	N	N	N	Y	Y	Y	Y	Y	Y	N	Y	N	NA	NA	NA	NA	NA	NA	NA	NA	NA	Y	N	Y
Developmental delay	N	N	N	N	N	N	N	N	Y	Y	Y	Y	Y	N	N	N	NA	NA	NA	NA	NA	NA	NA	NA	NA	N	N	Y
Umbilical hernia	Y	N	N	N	N	Y	N	Y	N	Y	Y	Y	Y	Y	NA	N	NA	NA	NA	NA	NA	NA	NA	NA	NA	N	N	N
Hepatomegaly	Y	Y	Y	N	NA	Y	N	Y	Y	Y	Y	Y	NA	N	N	N	NA	NA	NA	NA	NA	NA	NA	NA	NA	Y	Y	Y
Splenomegaly	Y	N	Y	N	NA	Y	N	N	N	NA	Y	NA	NA	N	NA	Y	NA	NA	NA	NA	NA	NA	NA	NA	NA	N	N	N
Fatty liver	Y	Y	N	Y	NA	N	N	NA	N	NA	NA	NA	NA	N	N	N	NA	NA	NA	NA	NA	NA	NA	NA	NA	N	N	N
Osteopenia	N	N	NA	NA	NA	NA	NA	NA	NA	NA	NA	NA	NA	NA	NA	Y	NA	NA	NA	NA	NA	NA	NA	NA	NA	N	N	N
Osteoporosis	Y	Y	NA	NA	NA	NA	NA	NA	NA	NA	NA	NA	NA	NA	NA	N	NA	NA	NA	NA	NA	NA	NA	NA	NA	N	N	N
Dolichosigmoid	Y	N	NA	NA	NA	NA	NA	N	NA	NA	NA	NA	NA	NA	NA	N	NA	NA	NA	NA	NA	NA	NA	NA	NA	NA	NA	N
Frequent infectious diseases	Y	Y	N	Y	N	N	Y	NA	NA	NA	NA	Y	NA	NA	NA	Y	NA	NA	NA	NA	NA	NA	NA	NA	NA	N	N	N
Vitamin D defficiency	Y	NA	NA	NA	NA	NA	NA	NA	NA	NA	NA	NA	NA	NA	NA	NA	NA	NA	NA	NA	NA	NA	NA	NA	NA	NA	NA	N
Hypocalcemia	Y	NA	NA	NA	NA	NA	NA	NA	NA	NA	N	N	NA	NA	NA	NA	NA	NA	NA	NA	NA	NA	NA	NA	NA	NA	NA	NA
Hyperuricemia	Y	NA	NA	NA	NA	NA	NA	NA	NA	NA	N	N	NA	NA	NA	NA	NA	NA	NA	NA	NA	NA	NA	NA	NA	NA	NA	NA
Bilateral cataract	Y	NA	NA	NA	NA	NA	NA	N	NA	NA	NA	NA	NA	NA	NA	NA	NA	NA	NA	NA	NA	NA	NA	NA	NA	N	N	N
Macroglossia	N	N	N	N	N	N	N	N	N	N	N	N	Y	N	Y	Y	NA	NA	NA	NA	NA	NA	NA	NA	N	N	N	N
Cutis marmorata	N	N	N	N	N	N	N	N	NA	NA	NA	NA	NA	NA	NA	Y	NA	NA	NA	NA	NA	NA	NA	NA	N	N	N	N
Constipation	Y	N	Y	N	N	N	Y	N	NA	NA	NA	NA	NA	NA	NA	N	NA	NA	NA	NA	NA	NA	NA	NA	N	N	N	N
Hearing loss	N	N	N	N	N	N	N	Y	N	N	N	N	N	N	N	N	NA	NA	NA	NA	NA	NA	NA	NA	N	N	N	N

*R* Russia, *J* Japan, *M* Mexico, *T* Turkey, *O* Oman, *SA* Saudi Arabia, *U* USA, *S* Sibling, *NA* Information not available, *F* Female, *M* Male, *Y* Yes, *N* No.

**Table 3:** Mutations in *CAVIN1* gene [5–14]

Patients	Mutations
Patient 1	c.631G > T:p.E211X(homozygous)
Patient 2	c.362dupT(homozygous)
Patient 3	c.696-697insC(homozygous)
Patient 4	c.696-697insC(homozygous)
Patient 5	c.696-697insC(homozygous)
Patient 6	c.696-697insC(homozygous)
Patient 7	c.696-697insC/c.525delG
Patient 8	c.135delG
Patient 9	c.135delG
Patient 10	c.481-482insGTGA
Patient 11	c.518-521delAAGA
Patient 12	c.518-521delAAGA
Patient 13	c.259C > T
Patient 14	c.512CNA /c.696_697insC
Patient 15	c.696_697insC
Patient 16	c.160delG (homozygous)
Patient 17	c.160delG (homozygous)
Patient 18	c.160delG (homozygous)
Patient 19	c.160delG (homozygous)
Patient 20	c.160delG (homozygous)
Patient 21	c.160delG (homozygous) с.45G > A (homozygous)
Patient 22	c.160delG (homozygous)
Patient 23	c.160delG (homozygous)
Patient 24	c.160delG (homozygous)
Patient 25	c.160delG (homozygous)
Patient 26	c.550G > T
Patient 27	c.550G > T
Patient 28	c.518521delAAGA and c.471 + 1G.T

Our patient had all the described signs except acanthosis nigricans. However, other metabolic and clinical manifestations, which were not found in other patients with this form of СGL, are noteworthy: diabetes mellitus, vitamin D deficiency, hypocalcemia, hyperuricemia, bilateral cataracts. Dolichosigmoid was not found in other patients with CGL type 4, but can be attributed to the disorders of gastrointestinal tract connected with this form of СGL, like hypertrophic pyloric stenosis and constipation.

The remaining endogenous insulin secretion (C-peptide level 0.9 ng/ml) is not typical for type 1 diabetes over an 18-year period. Furthermore, for 2 years the patient was on a diet, without insulin therapy, with no history of ketoacidosis. Anti-insulin antibodies are positive (17 U/ml), which can be a result of a long-term insulin therapy, the rest of the immunological markers for type 1 diabetes are negative (Table 1). Thus, it is more likely that the patient has lipoatrophic diabetes rather than type 1 diabetes, despite the lack of evidence of insulin resistance (no acanthosis nigricans, insulin 60 U/day, insulin resistance indexes assessment was compromised due to a long-term experience of diabetes mellitus, and a wound defect of the right thigh). Nevertheless, insulin therapy is a front-line therapy for this patient because of the concomitant diseases.

Vitamin D deficiency and hypocalcemia, found in our patient, was previously found only in CGL type 3. However, osteoporosis was reported in CGL types 3 and 4 and is typical for progeroid syndromes which are also associated with generalized lipodystrophy.

Bilateral cataracts were not previously described in any CGL forms, but they are a typical sign of Werner syndrome (“progeria of adults”) [15].

Hyperuricemia has not been reported in CGL, however it was found in familial partial lipodystrophy type 3 caused by *PPARG* mutation [16] In addition, it is a frequent component of metabolic syndrome.

In generalized lipodystrophy, metreleptin (with diet) is a first-line treatment for metabolic and endocrine abnormalities and may be considered as a prevention of these comorbidities in children [1].

## Conclusions

In comparison with previously reported patients with CGL type 4, our patient has insulin dependent diabetes mellitus, vitamin D deficiency, hypocalcemia, bilateral cataracts, hyperuricemia. All these manifestations are known to be associated with other lipodystrophy syndromes, but to our knowledge it is the first time they have been shown to be associated with CGL type 4.

## Data Availability

Not applicable.

## Abbreviations

BMI: Body mass index
CGL: Congenital generalized lipodystrophy
CT: Computed Tomography
